# Workforce planning, staffing and education requirements for radiation therapists (RTTs) in the European Union as part of the EU-REST project

**DOI:** 10.1016/j.tipsro.2026.100403

**Published:** 2026-04-20

**Authors:** Mary Coffey, Michelle Leech, on behalf of the European Society for Radiotherapy and Oncology

**Affiliations:** a Applied Radiation Therapy Trinity, Discipline of Radiation Therapy, Trinity College Dublin, Ireland; b Trinity St. James’s Cancer Institute, Dublin, Ireland

**Keywords:** Workforce planning, Education and training, Policy formation

## Abstract

**Background:**

The European Society for Radiotherapy and Oncology (ESTRO), together with other relevant European societies, took part in a recent project focused on workforce availability, education and staffing in medical applications of radiation in the European Union (EU). The required output of the project was to define policy guidelines for the EU on appropriate education and training and workforce planning standards for professionals working in medical applications of radiation.

**Methodology:**

Data on workforce and education and training across specialties in the EU were collected using pre-survey and survey methodologies. The surveys were sent to national organisations and competent authorities, national professional societies, national radiation protection authorities through the Heads of the European Radiological Protection Competent Authorities (HERCA) and national medical associations/chambers through European Union of Medical Specialists (UEMS).

A scoping literature review on both workforce planning and education and training requirements for all specialties was also conducted to inform the policy guidelines.

**Results:**

In this paper, we report on the results and policy recommendations on education and training (with a specific focus on radiation protection) and workforce planning for radiation therapists (RTTs) in the EU. The project highlighted the limited radiation therapy-specific content in the education of radiation therapists across the EU, with some notable exceptions. It also highlighted the absence of workforce planning for radiation therapists, which is likely contributing to the current shortfall both in the EU and globally.

**Conclusion:**

RTTs are core members of the radiation oncology team and treatment cannot be delivered in their absence. From this project, it is recommended that the EU prioritise education and training for radiation therapists with a particular separation on radiation protection education from other specialties. Activity-based workforce modelling for radiation therapists is recommended to flexibly accommodate the varied roles and responsibilities of RTTs across the EU.

## Highlights

• This paper reports on the radiation therapist results of the EU-REST project.
• The EU-REST project was focused on guidance to the EU on education and workforce planning for those using ionising radiation.
• Specific radiation therapy content is limited in many education programmes.
• A lack of workforce planning for radiation therapists is apparent.
• Activity-based workforce planning modelling is optimal.

## Introduction

In 2022, the European Society for Radiotherapy and Oncology (ESTRO) was part of a consortium that was awarded a tender by the European Health and Digital Executive Agency (HaDEA), acting under a mandate from the European Commission’s Directorate General for Health and Food Safety (DG SANTE) in collaboration with the Directorate General for Energy (DG ENER). The other consortium members were the European Society of Radiology (ESR) (lead partner), the European Federation of Radiographer Societies (EFRS), the European Association of Nuclear Medicine (EANM) and the European Federation of Organisations for Medical Physics (EFOMP). The focus of the project was current workforce availability, education and staffing in medical applications of radiation within the European Union (EU). The output of the project was to define policy guidelines for the EU on appropriate education and training and workforce planning standards. This paper focuses on those policy guidelines for radiation therapists (RTT), consistent with those professionals defined under the European Skills, Competences, Qualifications and Occupations (ESCO) framework [1]. This project was particularly important given the current significant shortage of well-educated and trained radiation therapists globally [2], [3], [4] and the lack of specialist education and training specific to radiation therapy [5], [6].

The overarching aims of the project were:

To gather and collate data on workforce availability and education and training needs (with a specific focus on radiation protection) to ensure quality and safety of practice.
Develop guidelines for workforce planning and education and training of individual specialties within the consortium.
Provide the EU with recommendations on workforce availability, education and training necessary to ensure quality and safe practice.

This paper will report on the results and policy recommendations on education and training and workforce planning specifically for radiation therapists (RTTs) in the EU.

## Methodology

**Fig. 1:**
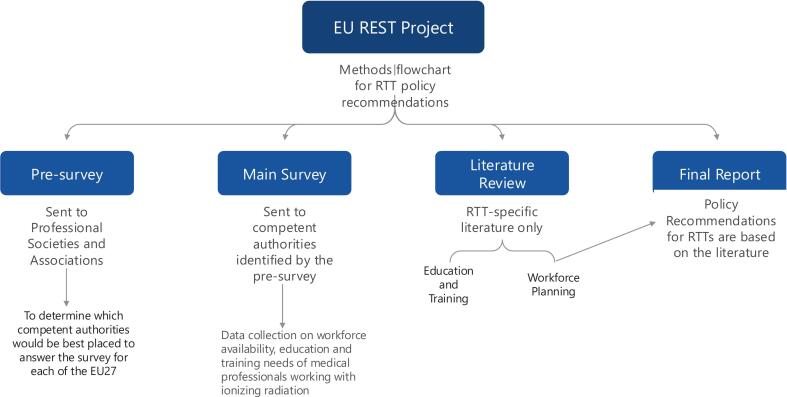
Methods flowchart.

As the title and associated competences of the profession of Radiation Therapist have been recognised by ESCO and adopted by ESTRO, it was decided that radiation therapist workforce and education and training requirements would be considered independent of diagnostic radiography and nuclear medicine. The project design has previously been published [7], but in short was comprised of four main components (Fig. 1).

First, a pre-survey was designed to ascertain which bodies would be best placed to provide data on workforce numbers and education and training requirements in each country. The consortium members ESR, EFOMP, EFRS and ESTRO, as well as Advisory Board Member EANM, distributed the link to the Pre-Survey, as did the EC to members of the SAMIRA Steering Group on Quality and Safety (SGQS). From this, 273 contacts from relevant authorities and professional bodies in the EU were sent the main survey.

The main survey of over 400 questions was developed and subsequently sent to those identified in the pre-survey i.e. national organisations and competent authorities, national professional societies, national radiation protection authorities through the Heads of the European Radiological Protection Competent Authorities (HERCA) and national medical associations/chambers through European Union of Medical Specialists (UEMS) via the SurveyMonkey platform (SurveyMonkey Inc. SurveyMonkey [Internet]. San Mateo, CA: SurveyMonkey; 2024 [cited 2024 Nov 6]. Available from: https://www.surveymonkey.com). The high number of questions in the survey was deemed necessary by the consortium to ensure that all professional groups were included. This survey was divided into four sections pertaining to education and training (including continuous professional development (CPD)/continuing medical education (CME), workforce availability and planning and quality and safety. Universities or other education institutes were not incuded in this survey given the high number of both involved in education and the different professions covered in the project.

A narrative literature review, encompassing national, European and international guidelines on education and training and workforce planning for radiation therapists was undertaken by the ESTRO team. The literature search focused on national/international guidelines at EU level, but guidelines from other, non-European countries with levels of healthcare systems like Europe were also considered. Grey literature issued by relevant societies/organisations such as the International Atomic Energy Agency was also included.

First, a search of PubMed/MEDLINE was conducted using a combination of Medical Subject Headings (MeSH) and free-text terms related to radiation therapists and workforce planning. Search terms included “Radiation Therapists”[MeSH] OR “radiation therapist*” OR “radiotherapy technologist*” OR.

“radiation therapy technologist*” OR “therapeutic radiographer*” OR RTT).

“workforce,” “staffing,” “manpower planning,” “health services needs and demand,” and related synonyms. Boolean operators were used to combine population and workforce concepts. Second, using similar methods, a search related to education and training of radiation therapists was conducted. Search terms included “Radiation Therapists”[MeSH] OR “radiation therapist*” OR “radiotherapy technologist*” OR.

“radiation therapy technologist*” OR “therapeutic radiographer*” OR RTT).

AND (“Education”[MeSH] OR “Education, Professional”[MeSH] OR “ Training”[MeSH] OR “Clinical Competence”[MeSH] OR “Curriculum”[MeSH] OR education OR training OR competence OR curriculum OR teaching OR learning OR “professional development”)). Both searches were limited to the English language. No date restrictions were applied.

Throughout the project there were multiple stakeholder meetings and feedback on the data produced. Finally, the guidelines for both workforce planning and education and training for each specialty were developed, based on the survey findings and the literature review.

## Results

A total of 186 responses to the survey were received with varying levels of completeness. Of these only 7% (n = 13) pertained exclusively to radiation therapists and were from 7 countries. The majority of responses received were from national professional or scientific societies rather than competent authorities. Given the paucity of data from the survey for radiation therapists, the information was deemed largely ‘non-useable’ in the project. Although radiographers were asked to indicate in the survey if they were responding on behalf of radiation therapists, there was no indication of this noted in many instances. The number of radiation therapists and radiographers per million inhabitants varied from 86 (Belgium) to 613 (Finland) with the EU average at 385. However, deciphering the specific number of radiation therapists was not possible given the data that were received in the survey. This was further compounded by the absence of inclusion of non-radiography professionals such as nurses who fulfil the role of radiation therapist in some EU countries such as Belgium and Sweden.

The duration of education and training reported from the limited survey results ranged from 0-4 years with the 4-year programmes (n = 2) offering dedicated radiation therapist degrees and the majority of courses (n = 11) joint with other disciplines, with minimal radiation therapy-specific content. There was a range of 0–30% clinical practice education required to work as a radiation therapist in the clinic.

Eleven papers were identified in the literature review that related to education and training for radiation therapists in Europe [5], [6], [9], [10], [11], [12], [13], [14], [15], [16], [17]. Two addressed specific aspects of radiation therapy practice [9], [10], one addressed the barriers to radiation therapist education, specifically in Belgium [11] and one compared radiation therapy curricula in selected EU countries [6]. Two papers benchmarked competencies at entry level and for advanced practice. Core curricula for radiation therapists have been developed by ESTRO [13] and the International Atomic Energy Agency (IAEA) [14] and curricula are recommended by professional societies in Australia [15] Canada and the United States and were included in the literature review. From this literature review, the following policy guidelines were developed (Box 1).

Box 1 **Education and Training Guidelines for Radiation Therapists**

**Table:** 

Education and training programmes should be sufficiently robust to ensure that when licenses are granted, graduates are competent to practice. This means that upon graduation, radiation therapists should be competent to work immediately in the clinic, without the need for additional education or extended internship or mentorship periods.
Education and training programmes should be sufficiently robust to ensure that when licenses are granted, graduates are competent to practice. This means that upon graduation, radiation therapists should be competent to work immediately in the clinic, without the need for additional education or extended internship or mentorship periods.
Education programmes should be 3 years (180 ECTs) dedicated to radiation therapy, with a minimum of 20-30% clinical training across a range of clinical settings. This programme duration ensures that the necessary theoretical knowledge of the physical and biological impacts of radiation is clearly understood and applied to clinical practice. Clinical educators should be radiation therapists themselves and formal assessment of specified competencies should be documented.
The leader of the education programme should be a radiation therapist and both academic and clinical content should be radiation therapist-led.
Radiation protection education for radiation therapy needs to be considered in its own context, independent of diagnostic imaging.

Sixteen papers were identified in the literature review that related to staffing for radiation therapists in Europe [18], [19], [20], [21], [22], [23], [24], [25], [26], [27], [28], [29], [30], [31], [32], [33]. Previously, the ESTRO Health Economics in Radiation Oncology (HERO) project reported that the number of radiation therapists per linear accelerator across 20 countries in the EU ranged from 2 to 6 [18]. The European Organisation for the Research and Treatment of Cancer (EORTC) recommended more than two radiation therapists per linear accelerator, which was consistent with the IAEA recommendations [23].

In the context of new technologies in radiation therapy resulting in reduced treatment volumes with an enhanced requirement for accuracy and safety, the increasing use of artificial intelligence in treatment preparation and delivery and other evolving radiation therapist roles, innovative approaches are required to effectively capture the workload of a radiation therapy department, outside of traditional treatment delivery on linear accelerators.

From this literature review, the following guidelines were developed (Box 2). It should be noted that any calculation methodology used must reflect the requirements for safe and accurate practice and the evolving roles and responsibilities that radiation therapists will be expected to take in the future.

Box 2 **Workforce planning guidelines for radiation therapists**

**Table:** 

Staffing levels can no longer be simplified into recommended number of radiation therapists per linear accelerator or computed tomography unit or patient number.
An activity-based model should be used that is sufficiently flexible to encompass the activity of individual departments in the EU including CPD. This model recognises the need to include periods of leave such as maternity, paternity, parental and holidays as well as sick leave.
Specific areas of practice for radiation therapists should be considered. These include: Clinical roles (treatment units, simulation suites, treatment planning, brachytherapy, clinical roles of advanced practitioners), research and innovation, quality and risk management, education, management and leadership.
To ensure sufficient staffing levels to be able to deliver a safe and accurate service, the activity-based model should also encompass the following:
A radiation therapist must never work alone during treatment delivery
The staffing requirement must be cognisant of national legislation on working acts which will country-specific
The current staffing number and status (full time, part time etc.) must be known
Working hours of the department and whether there is a shift system in place must be noted
Scheduled maintenance, downtime and equipment replacement programmes must be included
For consistency of service in the future and to inform education institutes of the potential future student intake, equipment and any planned extension, evolving staff roles and responsibilities and attrition and retirements must be considered

## Discussion

The paucity of results to the survey reflects the inability of the national societies and organisations as well as the competent authorities to adequately answer the radiation therapist component, as well as likely deterred by a lengthy survey. The limited responses received confirmed the significant lack of academic and clinical education for radiation therapists across the EU, and this was corroborated by the literature review. Previous research in education and training of radiation therapists [6], [13] reported that in most European countries the radiation therapy component of education programmes is less than 20% of the overall curricula and, in some instances, there is no requirement at all to include radiation therapy.

As EU legislation thus far has failed to separate radiation therapists from radiographers [8], detailing the workforce available to radiation therapy in Europe in this survey resulted in limited useful data, however it is well documented that there is currently a global shortage [4], [34]. It became apparent from the literature that there are limited workforce models specific to radiation therapists in use within the EU. The guidelines in this paper are based on the literature available in the area namely from the ESTRO HERO project, the IAEA staffing model and published Canadian workforce models [18], [23], [21].

For the education guidelines with respect to radiation protection, published literature in the area was used and additional content was added to reflect the significant changes in radiation therapy practice in the modern era. The additional material related to the need to understand the biological as well as the physical impact of radiation and awareness of dose constraints to organs at risk under different treatment conditions. Skills training to carry out specific tasks is not sufficient for modern quality and safe practice.

Separation between the medical radiation professions with respect to radiation protection at legislative level has been identified through working on the EU-REST project. For future EU research projects, a specialty-specific approach is essential if data collected are to be useful and informative. This was demonstrated by the length of the survey necessary to incorporate all disciplines and professionals and the very poor results relating to the radiation therapist particularly.

The current legislation [8] which influences education and training programmes in determining the radiation protection content has contributed to the paucity of radiation therapy-specific education across the EU as it defines the radiation protection requirement of education programmes. The legally required content is generic failing to recognize the need for radiation therapists to understand the biological effect of high dose radiation on normal tissue. Diagnostic imaging is frequently a discrete or a series of discrete events with a much lower associated dose than radiation therapy, which typically extends over a defined period of time delivering much higher doses. Therapeutic doses can be biologically equivalent up to 200 Gy in comparison to the average dose for an abdominal CT of 10–20 mGy. Because of this, errors in the delivery of radiation therapy can have significant clinical consequences for patients. Minimising such errors is a critical component of radiation protection and patient safety and should be reflected in the legislation.

## Conclusion

European legislation on radiation protection should reflect current and future radiation therapy practice independent of diagnostic imaging and nuclear medicine. The ultimate goal is the optimum treatment of patients receiving radiation therapy and this cannot be achieved if RTTs, who are responsible for preparation and treatment delivery have not received appropriate education and training. Workforce planning for radiation therapists must be considered across the EU using appropriate modelling such as activity-based modesl which are flexible for the varied roles and responsibilities of radiation therapists in EU member states.

## Funding

The EU-REST project was funded by the EU4Health Programme of the EU under service contract HADEA/2022/OP/0003 with the European Health and Digital Executive Agency (HaDEA).

## Declaration of competing interest

The authors declare that they have no known competing financial interests or personal relationships that could have appeared to influence the work reported in this paper.
